# Adults With Neurocognitive Disorders Who Reside Alone: Exploration of Nontraditional and Absent Support Systems

**DOI:** 10.1093/geroni/igaa057.2023

**Published:** 2020-12-16

**Authors:** Laura Girling, Kate de Medeiros

**Affiliations:** 1 University of Maryland, Baltimore County, Baltimore, Maryland, United States; 2 Miami University, Oxford, Ohio, United States

## Abstract

Research steadily demonstrates that family functions as the central component in the provision of care for persons with neurocognitive disorders. While it is clear family plays a critical role in the lives of adults with neurocognitive disorders, overlooked is the subpopulation who reside alone, but have no identifiable family to provide care. To address this gap, data were drawn from an interview-based NIA-funded study that focused on community-dwelling live-alone persons with dementia. Subanalyses were conducted on the interviews and field notes of live-alone adults with neurocognitive disorders who had no identifiable family (N=19) and their collaterals (e.g., neighbor, N=20). Using data-derived coding in ATLAS.ti., several themes emerged including transient informal care, consequential peripheral ties, and strained/traumatic nuclear relations. Themes will be discussed in detail. The present study expands the limited information on community-dwelling persons with dementia, providing a lens for understanding the complex intersection of aging and non-traditional/absent support networks.

